# Strategies and tensions in communicating research on sexual and reproductive health, HIV and AIDS: a qualitative study of the experiences of researchers and communications staff

**DOI:** 10.1186/1478-4505-9-S1-S4

**Published:** 2011-06-16

**Authors:** Joanna Crichton, Sally Theobald

**Affiliations:** 1 African Population and Health Research Center, PO Box 10787, 00100 GPO, Nairobi, Kenya. Current address: School of Social and Community Medicine, University of Bristol, Canynge Hall, 39 Whatley Road, Bristol, BS8 2PS, UK; 2 Dr. Sally Theobald, International Health Research Group, Liverpool School of Tropical Medicine, Pembroke Place, Liverpool, L3 5QA, UK

## Abstract

**Background:**

Sexual and Reproductive Health (SRH) and HIV issues are often controversial and neglected, leading to challenges with engaging policy actors. Research evidence is complex, posing further challenges for ensuring that policy and practice are evidence-based. Many health researchers are adopting innovative approaches to engaging stakeholders in their research, yet these experiences are not often shared. This qualitative study focuses on the research communication and policy influencing objectives, strategies and experiences of four research consortia working on SRH, HIV and AIDS.

**Methods:**

We carried out 22 in-depth interviews with researchers and communications specialists (research actors) from the four consortia and their partners, working in nine countries in sub-Saharan Africa and Asia. Using the ‘framework’ approach to qualitative data analysis, we identified factors that affect the interaction of research evidence with policy and practice. We used the ODI RAPID analytical framework to present these results, adapting this tool by incorporating the actions, strategies and positionality of research actors.

**Results:**

The characteristics of researchers and their institutions, policy context, the multiplicity of actors, and the nature of the research evidence all play a role in policy influencing processes. Research actors perceived a trend towards increasingly intensive and varied communication approaches. Effective influencing strategies include making strategic alliances and coalitions and framing research evidence in ways that are most attractive to particular policy audiences. Tensions include the need to identify and avoid unnecessary communication or unintended impacts, challenges in assessing and attributing impact and the need for adequate resources and skills for communications work.

**Conclusions:**

We contend that the adapted RAPID framework can serve as a tool for research actors to use in resolving these tensions, through facilitating a reflexive approach to considering their own combination of attributes, skills, networks and objectives and the ways these relate to policy contexts, actors and processes.

## Background

Research-to-policy interactions are not straightforward and research findings are not a passport to policy [1-4]. In the past decade, many donors and research institutions have placed increased emphasis on research communication and translating evidence into policy and practice [5-7]. Some donors have increased their expectations for the kinds of influencing objectives that researchers should achieve. Recent pressures on academic budgets may increase scrutiny about research impact at the same time as potentially reducing resources available for communication [8]. This necessitates further understanding and action on how to ensure intensified research communication and policy engagement activities are strategic, appropriate and effective.

The ‘how to’ of research that influences policy and practice is hotly debated. However, most research focuses on resource rich countries and surprisingly little empirical or theoretical analysis from health research in resource-poor contexts [9]. Gilson and McIntyre [10] argue that there are limited formal conceptual or empirical analyses of the research policy interface in the health sector and that ‘The mechanisms and processes through which research impacts on health policy remain cloudy’.

The subject matter of research is critical to consider, because policy influencing processes may vary with different issues and types of evidence. This study focuses on the arena of sexual and reproductive health (SRH), HIV and AIDS, which is fast moving, multidisciplinary and involves multiple stakeholders working at different levels. The issues can be sensitive and challenging and the messages and processes through which to engage with diverse stakeholders in different contexts need careful consideration and further empirical and conceptual understanding.

The literature on the research-to-policy interface is fast growing, and a number of studies have been carried out reviewing the role of research evidence in policy processes, including the Overseas Development Institute (ODI) Research and Policy in Development (RAPID) programme[9,11,12]; International Development Research Centre (IDRC) [13-15]; and the Economic and Social Research Council (ESRC) [16], and the World Health Organisation’s Health Research Systems Analysis (HRSA) Initiative [7]. These research programmes have generated a number of different models of the research to policy processes, as explored by Sumner et al. in another paper in this supplement [17] Less common in this growing literature is reflection from researchers or research-funding organisations themselves about the ideal roles of research organisations, about the compatibility between research and communications objectives, and about some of the tensions and challenges involved in policy influencing. As demonstrated by Walt et al. [18], researchers need to engage with their own positionality in policy processes both to understand and to influence policy processes.

In this paper, we use a participant-observer approach to carry out collaborative analysis with researchers and communicators in four research programme consortia (RPC) working on sexual and reproductive health (SRH). We used in-depth interviews, case studies and an interactive workshop to explore these actors’ views on the role of research in policy, and their experiences with policy engagement. We draw on the RAPID analytical framework in ordering and presenting our results. We adapt this framework by adding a sphere on the characteristics and actions of researchers and their institutions.

Our paper contributes to knowledge about the research-to-policy interface in two ways. Firstly, we examine the policy engagement experiences of research actors working on SRH and HIV/AIDS across a variety of contexts, types of research organisations and research methods. Secondly, we highlight the importance for research actors to reflect on their own positionality in policy processes and the implications for their research strategies, demonstrating how an adapted version of the RAPID framework can be used for this purpose. Finally, we identify and share experiences and lessons for enhancing the impact of research on health policy and practice.

There are various terms to describe the key actors in the research-to-policy interface. In this paper we use two terms to group together those who produce and communicate research on the one hand and those they are seeking to inform or influence on the other. Firstly, we introduce the term ‘research actors’, which we use to refer collectively to both researchers and professionals who specialise in communicating research. Secondly, we use the term ‘policy actors’ as a collective term for the target audiences for most health research. Following Gilson and Raphaely [19], we define policy actors as all those who have a stake in health policy, including domestic and international actors who make or help shape decisions about health policy and practice in government, not-for profit and for-profit institutions. Finally, we use the term ‘intermediaries’ to refer to secondary audiences such as media or advocacy groups, who may take up research evidence and communicate it to other policy actors.

## Methods

We approached the study in three ways: as a piece of research, as a process of reflection about our research and policy engagement work and as an opportunity to exchange ideas and lessons. As research authors working in RPCs, we are also participant observers in the institutions and processes that we are studying and undertook a reflexive approach to interviewing and analysis [20]. The RPCs we selected are listed in Table 1. Our reasons for selecting these RPCs were because they were all the RPCs working on SRH and HIV that were funded by a single donor, the UK Department for International Development (DFID) and that they represented varied types of research organisations (policy-oriented, academic, and service delivery) working in multiple countries and at national and international influencing levels. Selecting this group of RPCs enabled comparative analysis of variations relating to policy contexts. In addition, it provided the opportunity for the partners in the consortia to share their experiences in addressing the communications objectives of a common donor.

**Table 1:** Research Programmes selected for study

Realising Rights: improving sexual and reproductive health for poor and vulnerable populations Institute of Development Studies (IDS), UK – lead partner BRAC, Bangladesh African Population and Health Research Center (APHRC), Kenya In-depth Network, Ghana London School of Hygiene and Tropical Medicine (LSHTM), UK
**Addressing the Balance of Burden in HIV/AIDS (ABBA)** Liverpool School of Tropical Medicine (LSTM), UK – lead partner Research for Equity and Community Health (REACH Trust), Malawi Regional AIDS Training Network (RATN), Kenya Health Research Unit (HRU), MoH, Ghana Health Economics and HIV/AIDS Research Division, University of KwaZulu-Natal (HEARD), South Africa Population Council, USA

**Research and Capacity Building in SRH and HIV in Developing Countries** London School of Hygiene and Tropical Medicine (LSHTM), UK – lead partner National Institute for Medical Research, Tanzania Navrongo Health Research Centre, Ghana School of Medical Sciences, Kwame Nkrumah University of Science and Technology, Ghana Reproductive Health and HIV Research Unit, University of the Witwatersrand, South Social and Public Health Sciences Unit of the Medical Research Council, (MRC) UK International Planned Parenthood Federation (IPPF) Population Services International (PSI)

**Evidence for Action: An International Research Consortium to Maximise Benefits & Equity of HIV Treatment & Care Systems** London School of Hygiene and Tropical Medicine (LSHTM), UK – lead partner International HIV/AIDS Alliance, in Brighton, UK Lighthouse Trust in Lilongwe, Malawi Medical Research Council UVRI Uganda Research Unit on AIDS, Uganda National AIDS Research Institute, India University College London and the Medical Research Council’s Clinical Trials Unit, UK Zambian AIDS-Related Tuberculosis Project (ZAMBART), Zambia

Between August 2008 and May 2009, we conducted 22 in-depth interviews (IDIs) with researchers and communications officers from northern and southern partners in the four RPCs (see table 2 for the interview breakdown). We used a combination of purposive and convenience sampling to identify individuals to interview, while ensuring a range of Northern and Southern communications and research staff from each RPC. The interviews were face to face wherever possible and in two cases over the telephone. An informed consent procedure was adhered to, including information on how the data would be used and the opportunity to review the draft paper. The interviews were conducted in English, transcribed, and imported into MAXQDA software. Following the framework approach to qualitative analysis [21] we coded the interviews according to themes and sub-themes, refining the codes and their definitions collaboratively during the process. Each text was coded by one of us and then reviewed by the other and further codes were added where appropriate. During analysis, we identified concepts and frameworks from the literature on the research to policy interface that resonated with the themes emerging from our data. We used this to select the ODI RAPID framework [9,11], which we identified as an appropriate analytical framework for grouping the emerging themes. We asked researchers and communications staff from each consortia to develop case studies of their experiences with policy engagement. We used these case studies to further contextualise and triangulate the themes emerging from the interview data.

**Table 2:** Breakdown of in-depth interviews

	ABBA	EFA	Realising Rights	SRH & HIV
Researchers, South	3	2	3	1

Researchers, North	1	0	3	2

Communications staff, South	0^1^	1	3	0^1^

Communications Staff, North	0^1^	1	1	1

^1^ No staff members matching this description were working for the consortium at the time the interviews were conducted.

The study was designed so that it included opportunities for researchers and communications staff in all the research consortia to provide feedback on the analytical framework and results. We held a stakeholder workshop in Liverpool on 18-19 May 2009, where we presented an earlier draft of the paper and the case studies were presented. Participants were given the opportunity to comment on the paper findings. The workshop enabled us to identify themes that had particular salience for participants. We then carried out another layer of analysis of the data and writing after receiving this feedback, and identified additional themes and relationships between themes.

The study was reviewed by and received ethical approval from the Liverpool School of Tropical Medicine Ethics Committee, reference number: 08.48

## Results

### Our conceptual framework

**Figure 1:**
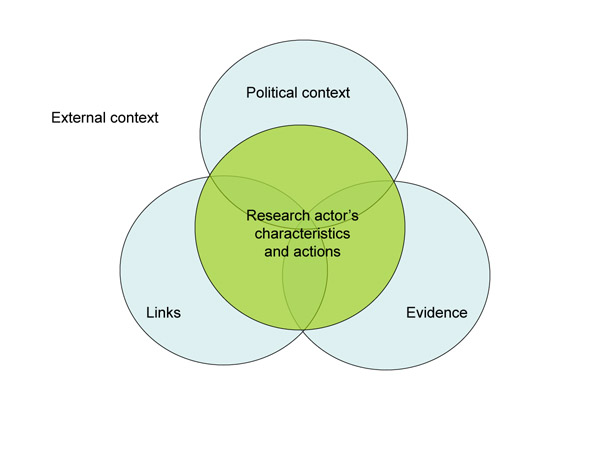
**The research-policy interface**. Figure adapted from Crewe, E. & Young, J. (2002). Bridging Research and Policy: Context, Evidence and Links ODI Working Papers 173, September 2002.

During our analysis, we identified five groups of factors that emerged from our data as important in affecting the nature of the interface between research and policy. The first four groups of factors corresponded to those in the RAPID framework, namely the role of politics, other contextual factors (such as extra-national influences such as foreign donors, and social and cultural factors), ‘links’ (the relationships between research and policy actors), and the nature of research evidence (see Figure 1). In addition, our focus on research actors’ experiences with policy engagement required us to bring researchers and communications specialists explicitly into our analytical framework. We therefore adapted the RAPID framework to include a fifth group of factors affecting policy engagement, the characteristics and actions of research actors. Our interviews reveal how the attributes, skills and positionality of researchers and communications specialists, and their perceptions about the role of research evidence in policy and practice, help to shape their approach to policy engagement and the nature of influencing processes.

We present our research findings in the following order: we begin by examining the characteristics of research actors and the implications of this for policy engagement. We then discuss contextual factors, linkages between research and policy actors and evidence. For each of these groups of factors, we begin with the outer aspects of the RAPID framework, and then focus in on the dynamic elements of research engagement at the centre of Figure 1, exploring how research actors engage with each of these groups of factors in their work.

### The positionality and skills of research actors

The characteristics of research actors and institutions and their engagement with the other groups of factors emerged as important in our interview data and are represented by the research actors’ characteristics and actions’ circle at the heart of Figure 1.

Firstly, there was huge diversity between individual research actors in terms of their policy engagement attitudes, goals and skills. Each researcher and communications specialist we interviewed had a unique perspective on what policy influence means, what activities are part of policy influence, and what should be the role of research evidence in policy. Differences in views were in part shaped by disciplinary affiliation and in part by professional identity and personal outlook. Research actors appeared to be divided on two key issues. The first concerns whether the role of researchers is mainly to produce knowledge for other stakeholders to take up and use versus those who see engaging with policy actors as central to their role. The second key difference among all research actors was about whether research institutions should engage in influencing and advocacy, or stick to informing policy and practice rather than influencing it in certain directions.

In some cases, researchers argued that research and advocacy involve very different skills and attributes.

‘You know many good researchers don’t make good advocates. Quite a different skill and I think that's very rarely recognised. A good academic is trained to […] state the […] cautions, the doubts, whereas those are fatal qualities for an advocate who has to simplify, dramatise, exaggerate.’ (Researcher, London School of Hygiene and Tropical Medicine (LSHTM), UK).

In other cases, researchers emphasised the goal of influencing policy and promoting SRH rights among disadvantaged populations as an integral part of their work.

‘‘I’m pushy and I think of everything as an opportunity and I don’t shut doors and I keep resisting […] I am always looking for ways [for there to be] something more than just research in a book. Because […] I work a lot with poverty and urban poverty and especially […] in urban slums, I think [in terms] of the rights perspective or the structural inequalities for poor people’ (Researcher, BRAC University, Bangladesh)

Secondly, at the organisational level, the characteristics of a research institute influence the policy relevance of its research, and its approach to engagement with policy and practice. All interviewees were reflective about how the raison d’etre, history, and incentive systems of their organisation shaped their room for manoeuvre in the policy environment. Approaches to research communication varied according to whether organisations had mandates in academia, policy analysis, advocacy or service delivery.

Many researchers explained that incentive systems within their institutions did not reward policy engagement. Northern researchers in particular stressed that policy engagement is not valued in the Research Assessment Exercise (RAE) which is critical to how their work is assessed and their professional advancement. For example:

‘[Policy influencing] is something that I am very committed to […] but […] I think as a researcher within UK academia it is not an easy balance, it’s not what I’m judged by.’ (Researcher, LSHTM, UK)

The institutional disincentives against devoting time to communications activities can create challenges for communication specialists in working with researchers. For example:

Working with academics that's quite challenging. […]Not all of them see the value of communications necessarily or even what communications work might be and also they are slightly fearful of it. Sometimes for good reason if you have been misquoted in the press then I’m sure you wouldn’t necessarily want to do that work again for example. So it is a challenge to try and sell the value of communications whilst doing it at the same time (Communications officer, UK)

On the other hand, some communications staff and researchers noted that proponents of intensifying research communications often use vague language and unnecessary jargon, which alienate some researchers. In some cases, this leads researchers to feel that communications work involves moving outside their professional role instead of realising that some of the activities that they regard to be core parts of their research constitute communications. For example, one northern researcher explained that s/he seeks to increase the capacity of the southern Non-Governmental Organisations (NGOs) s/he works with to interpret research for policy, or to reduce prejudice against stigmatised groups held by some of her partners, but s/he had not previously identified this as communications work.

Building up cultures of mutual support amongst communication specialists and between researchers and communication staff can help to address these challenges. Respondents from one consortium described how they have established a community of practice, which acts as a supportive structure for those working on communication to share ideas and experiences. Another researcher described the contribution that support from communications staff makes to his/her work:

‘The role of our research communications officers has changed the way that we as researchers [...] relate to influencing work, policy work. They are experts at that and without their help I think we would still be producing little interesting consultancy reports or publishing articles, but now its much more diverse, strategic and proactive' (Researcher, IDS, UK)

### Context and strategic opportunism

The research actors we interviewed described how policy context and processes play an important role in determining the attitudes of policy actors to research evidence, their capacity to understand and use it and their scope to make or implement policies in response. Our findings revealed diverse political influences on uptake of SRH, HIV and AIDS research in different contexts.

‘I think quite often it’s serendipity whoever’s managed to get the attention of the policy maker at that particular time. I think there are lots of reasons why policy makers pick on a particular area and agree to fund a particular intervention and it may be that it’s due to a startling piece of RCT evidence that shows that the intervention works, but quite often it’s about the political profiling of that particular disease.’ (Researcher, LSHTM, UK)

A repeated theme from interviews with researchers working on SRH and HIV/AIDS was how attitudes to sex and sexual rights have an important influence on the uptake of policy in their field. Compared to many other research areas, cultural and social factors are strongly influential in political processes in this area. A researcher in Ghana described how moving into the area of AIDS has forced him to engage with societal attitudes to sexual relationships in a way that he never had to working on other, less sensitive, health issues.

‘Being a doctor I know what HIV is but in terms of the nuances and the politics and the understanding what to say, when and all that, I have been on a learning curve myself […] it’s been a little more challenging than the regular areas that I have been involved in […] In the African environment, issues about sex and sexuality are not discussed in the open […] and there is the need for us to get those issues addressed appropriately.’ (Researcher, Health Research Unit, Ghana Health Service)

Our interviews also demonstrated that engagement in policy processes varies by geographic scale, from district, provincial, national and international levels, with some research actors we interviewed focusing on one level and others working at all four simultaneously. In Zambia and Malawi, decentralisation in the health sector has led research actors to increasingly seek to influence district-level decision making and practice. Our findings show that international factors play a central role in national policy formation. A number of researchers from the north and south spoke about the importance of World Health Organisation (WHO) guidelines in influencing policy at the national level. A researcher from Ghana mentioned that the Ghanaian government is unlikely to make use of their research findings until they have been incorporated into WHO guidelines.

Moving into the central sphere of our conceptual framework, the research actors we interviewed described how their understanding of their political context affected their policy engagement strategies. Many of those we interviewed acknowledged that policy trends and financial flows can change direction quickly and suddenly, yet influencing policy often takes a long time, so policy direction can change before you have assembled the necessary evidence for influencing. Historical timing and serendipity therefore become important, as both communications staff and researchers talked about the importance of identifying and seizing opportunities. The combination of long processes of engagement and sudden points of change is illustrated by one communications officer:

‘… [ in some cases] entry points for influence are ignored by academics who think just because you have the evidence something will happen and actually it’s a very long process of forming relationships of trust, understanding how legislation’s brought in, how policy is brought in, and that happens sometimes very quickly and swiftly.’ (Communications officer, IDS, UK)

Some researchers and communications offices felt they had insufficient understanding of the policy processes they were trying to influence, because of lack of time to track policy, or insufficient policy analysis skills in their organisation. Some stated that they would like to develop a more sophisticated understanding of the policy processes they aim to influence, and engage in policy analysis.

‘It was very hard for me in my work to influence policy unless I had an understanding of the […] policy environment […] So I am changing the way I look at communication […] [You can’t] make much impact in influencing policy unless you understand what [policy makers] do, their challenges, their information needs.’ (Communications officer, African Population and Health Research Center (APHRC), Kenya)

### Links: Networking and coalitions

Interviewees had varied opinions on who constitutes a policy actor, depending on their context, professional/institutional goals and subject area. Some saw their target policy actors mainly as MoH or AIDS control council officials and parliamentarians at the national level and the WHO and donors at the international level. Others saw fellow researchers, public and private service providers, health campaigners, NGOs such as women’s advocacy groups, communities and their leaders, the media and health practitioners as also critical.

The field of HIV was reported as involving increasing complexity of actors, requiring new approaches to networking and coalition building. In many contexts, concurrent horizontal (Sector Wide Approaches or SWAp) and arguably more vertical approaches to health delivery such as the Global Fund to Fight for AIDS, TB and Malaria and the President’s Emergency Plan for AIDS Relief (PEPFAR) were also complicating the policy picture and the number and influence of policy actors. This trend was seen as both increasing the challenges of policy influence and creating new opportunities. Frequently changing ministers and parliamentarians also pose problems in fostering relationships with key national policy actors.

The links between research and policy actors varied widely both within and between consortia. Some participating RPC research organisations are *institutionally embedded* within the policy making process (for example the Research Development Department in the Addressing the Balance of Burden in HIV/AIDS RPC (ABBA RPC) is part of the Ghanaian MoH and BRAC University in Bangladesh (RR RPC) is formally separate from but closely linked to BRAC, a key player in service provision and policy making. Others are *intensely connected* to key policy actors and policy processes. For example the Lighthouse, in the Evidence for Action RPC (EFA RPC), has a unique working relationship with the Malawian MoH, as a service provider, partnering joint antiretroviral therapy (ART) monitoring activities, and as an adviser. Research for Equity and Community Health (REACH) Trust in Malawi (ABBA RPC) also enjoys very close working relationships with the MoH, sometimes conducting research in partnership. Other organisations are building linkages with policy actors because their existing channels of influence are less strong or routine.

Most interviewees from northern institutions largely constructed their approach to policy engagement as *supportive/strategic* in that they support southern institutions in policy engagement and seeking opportunities to exert influence at an international level. For example researchers from LSHTM (SRH/HIV RPC) have played a key role in developing the WHO Global Strategy on Sexually-Transmitted Infection (STI) Prevention and Care, the WHO STI Management Guidelines and the WHO global strategy on the Elimination of Congenital Syphilis, whilst carrying out research to inform these global policies in countries like Tanzania, Ghana, South Africa and Malawi.

Moving to the central sphere of Figure 1, we examine how researchers strategise to strengthen these linkages. Our findings show a clear paradigm shift amongst researchers and communication specialists alike from seeing research engagement as an important activity at the end of the research cycle to seeing research engagement as a critical activity throughout the research cycle. Consulting policy actors early on and building relationships throughout the research cycle helps to increase the policy-relevance of research and the receptiveness of policy actors to the research findings.

‘If you do [research] in partnership with government they easily accept the findings and take it up. […] if they were our partners from the very beginning of that project then they would easily believe its credible.’ (Communications Officer, APHRC, Kenya)

However, the intensity and frequency with which this engagement occurs varied between interacting with policy actors at specific researcher-defined stages in the research process, to continuously engaging in policy processes. The time commitments for the latter approach are particularly huge.

‘My experience in Malawi is that often being at the right place at the right time is what really opens the door to increased involvement […] so for us it’s not a matter of planning a set date on which we are going to interact with the MoH and give a presentation, its sitting there at the table all year long and then when you see that moment arise in a meeting you can put your foot in the door.’ (Communications officer, Lighthouse, Malawi)

Partnering with policy actors in the design and implementation of research projects increases their capacity to use the research and interpret the findings. This kind of engagement is clearly strategic but also a challenging investment involving time and resources. Given the intensive continuous forms of engagement needed for policy engagement, Southern research institutions and researchers based within them face particularly tough choices between academic achievement and policy influence.

‘Many research projects don’t put as much money into communications. For the most part it’s just like at a dissemination seminar and it ends there. Yet, policy engagement has to go far beyond that. […] you have to make sure that researchers are equipped to disseminate or to communicate effectively but beyond that is also the issue of what sort of resources are available to get that done.’ (Researcher, APHRC, Kenya).

Those institutions ‘intensely connected’ to key policy actors were also exploring ways to maintain and intensify these relationships – for example the Lighthouse spearheaded research on the feasibility of shifting responsibility for ART provision to Health Surveillance Assistants after a request from colleagues in the Malawian MoH. Less well-connected institutions are seeking to build mutual understanding, trust and relationships through repeated, informal interaction, and building the demand for and capacity to use research evidence among policy actors and the media.

A recurrent observation by both researchers and communications staff is that they consciously make strategic alliances with other influential or mediating actors in order to more effectively access the rapidly changing policy arena. Research actors base their decisions about which kind of alliances to foster on strategic opportunities afforded by the policy context and their level of access to policy audiences, as explored in another paper in this supplement [22]. Developing collaborative relationships with influential spokespersons was found to be another effective form of strategic alliance, for example HEARD in South Africa have enhanced their influence through working with a mayor who has ’championed’ their research in provincial policy processes.

### Evidence, messages and rallying ideas

Research actors we interviewed regarded the type of evidence engagement strategies as having important implications for the most appropriate approach to communication. Some researchers stressed the critical importance of basing communications strategy on careful analysis of what should be the scope and limitations of the research findings, and therefore the type of influence it should have on policy. For example, clinical trial evidence can be particularly challenging for policy engagement because no one study is likely to be definitive, results are often complex and can appear contradictory. An appropriate use of the research findings is often more for contributing to the state of knowledge in an area, rather than for influencing policy directly based on the results of one clinical trial. Researchers also noted the importance of considering the appropriate scale of influence, for example the importance of not trying to influence international policy with research findings that are only relevant to a specific context.

‘Thinking in terms of my medical background of a researcher, first you don’t want to give them any false hopes […] That’s the difference between trying to change policy when you believe you have very strong evidence and wanting to change policy for the sake of it[…] If this proves to be detrimental to populations, I think this is the biggest fear.’ (Researcher, UK)

Examining the inner circle of Figure 1, we identified a number of strategies research actors in the field of SRH and HIV used to try to bring their evidence alive to different policy players. Southern communication specialists at APHRC and the INDEPTH Network highlighted the importance of ‘immersion’, where researchers take policy actors or intermediaries to a research site to encourage them to react to the issues on an emotional and experiential level. A perception from some that different types of research evidence may or may not resonate with different groups of policy actors was increasingly leading to ‘mix and match’ strategies whereby quantitative or costing data was presented alongside qualitative testimonies, photos, participatory maps or stories from the field.

Other researchers argued that from a policy actor’s perspective, methods matter less than perceptions about the credibility and legitimacy of individual researchers and research organisations and the quality of research. For some this led to increased attention to reflexivity and voice – i.e. who should present at which fora, in order to achieve maximum credibility and impact for the research. One northern researcher commented:

‘At the national level I would always take a step back, I think it is the prerogative and actually the duty of the national researchers to be the people at the forefront, they are the credible people, they are the ones to their national government’ (Researcher, UK).

Researchers and communications experts face challenges and dilemmas in framing their research findings for policy audiences and in part shape their strategies depending on whether their research focuses on areas constructed as mainstream, contested or neglected. Strategies include developing relationships and strategic alliances with a wide spectrum of policy actors and practitioners, and strategic use of concepts and buzz words targeted to specific audiences. Table 3 contains some illustrative examples.

**Table 3:** Influencing strategies

Creating public discussions of contested and neglected issues:	BRAC has worked to change attitudes of key government and media stakeholders towards sexuality in Bangladesh. This included holding a public meeting on sexuality, careful and active recruitment of participants, and capitalising on credibility held by academic institutions in Bangladesh to ally with queer groups. (*See: Rashid et al.*, *this issue*)
**Reframing contested issues:**	APHRC’s presentation of the results of their Protecting the Next Generation project at two international conferences, the first in Tanzania in 2007 and the second in Nigeria in 2008. The first time the information about the early age at which young people are starting to have sex and the need to provide them with SRH information and services was met with concern and anger by audiences, and the second time they presented the same messages in a more sensitive way (e.g. emphasising the importance of parents in young peoples’ lives and the dilemmas parents face) but with the same policy conclusions, the audience had a much more favourable reaction.

**Highlighting neglected issues:**	Bringing work on congenital syphilis higher up the policy agenda in Ghana through collaboration with the Ghanaian MoH. Generating buy-in for research findings at the national level through working with WHO on STI treatment and management guidelines (LSHTM and SRH and HIV partners).

**Extending or shifting mainstream issues**:	Strategic use of high profile conferences on relevant subjects. For example Evidence For Action (UK) held a satellite meeting at the XVII Mexico AIDS conference to share experience of using and generating evidence to improve HIV treatment and care systems in resource-poor settings. A communications officer from IDS (UK) sent out a blog from this meeting highlighting links between unfolding HIV debates and research from the Realising Rights consortium.

Some researchers actively sought to link their research findings to key terms or concepts that they felt would resonate with different policy actors. The Millennium Development Goals emerged as important here, as did terms such as human resources for health and scale up, rights and ‘balancing health and rights’. In some contexts, researchers and policy actors explained that they avoided the term ‘rights’ with some policy audiences because of policy actors’ suspicions about rights and viewing them as a foreign agenda.

‘Rights-based language has been used in the education sector and it has worked very well in Kenya. When it comes to health […] the challenge is […] when you mention the right to […] reproductive health then policy makers think abortion, policy makers think homosexuality and they just switch off […] I think the big thing would be […] sensitising them on what is a rights based approach to health.’ (Communications officer, APHRC, UK)

In some cases research actors explained that some ways of framing evidence are more likely to be acceptable to target audiences. (See Table 3 for a description of the way APHRC reframed findings on young people’s sexual health needs to make them more acceptable to their audience without losing their key messages.) Many researchers also faced challenges in summarising and or simplifying their research for different audiences and stated that they need further support in this endeavour. Others emphasized the hazards in over-simplifying research findings:

‘There is a tension and I do think that distilling of messages has to be done quite skilfully, otherwise you can have very negative unintended consequences.’ (Communications officer, Institute of Development Studies (IDS), UK)

‘I know that is not what policy actors want, they want to have a very clear-cut message, it’s so hard to give clear-cut messages because science is complicated and it’s always knowledge in progress.’ (Researcher, UK)

This point relates to one of the key distinctions among researchers between those who aim to use their research findings to *influence*, and those who aim to *influence knowledge as progress*. In the SRH/HIV and AIDS field, some researchers consciously use their research to try and change policy or academic discourses that they view as problematic, for example one Realising Rights RPC researcher is trying to influence prevailing discourses among donors about the impact of HIV on children and on gender and masculinities. Some organisations reinforce positive discourses by consistently promoting a message, for example the REACH Trust tries to continuously promote the concept of equity in health. An ABBA researcher is trying to influence health economics methodologies and policies, to think more broadly about quality of life and to use qualitative research as well as cost-benefit analysis:

‘I’m still pushing for quality of life tools to [be used in research and policy] since you are asking the individual how do they see the changes to their quality of life from receiving antiretroviral treatment or whatever intervention is been evaluated and sometimes […] it’s not only the treatment that makes a difference but […the fact] that they manage to get a job and [can] pay for things for children.’ (Researcher, University of Liverpool Management School, UK)

Choices about whether to approach policy engagement as influencing or knowledge as progress depend to a large degree on the type of research. Research evidence that supports a general shift in the way things are thought about or approached lends itself well to advocacy, whereas research that is more specific and incremental may be better suited to add to a pool of knowledge on an issue that can then be synthesized for policy change. For example:

‘The first part of my career I was involved in a huge project to map the demography of the world. What […] fertility was, what the contraceptive use in marriage might be, and those facts sort of spoke for themselves. […]There was no direct policy message coming from them but to make policy decisions in their absence would be inadequate and unsatisfactory and I think a lot of research is like that.’ (Researcher, LSHTM, UK)

A number of researchers and communications staff emphasised the importance of repetition of messages. A communications officer at IDS in UK described the effectiveness of communicating the same message repeatedly through multiple channels. A researcher at REACH Trust in Malawi said that s/he has influenced policy makers to think more about social aspects of health problems and services including health equity by consistently and repeatedly communicating a message:

‘It’s both really being consistent in your message and also engaging with the policy makers time and again and reinforcing the message that you are trying to bring on board.’ (Researcher, REACH Trust, Malawi)

## Discussion

Health research actors are seeking to influence health policy and practice that are extremely complicated and fast changing and are challenging to control, influence and measure. Despite the evidence-based policy agenda, the influence of SRH, HIV and AIDS research evidence is often eclipsed by other kinds of information and interests [1,23]. In order to influence policy and practice, research actors must interpret the policy context and the policy implications of their work. As Sumner et al [24] point out, the research actors we interviewed also have to make strategic decisions about three areas, firstly, identifying opportunities for influence within policy processes; secondly, forging strategic relationships and alliances, and thirdly, developing and articulating messages that are attractive to target audiences. As found in other studies, the research actors we interviewed who described themselves as working on neglected or contested policy issues found it useful to reframe research evidence in ways that are more resonant to policy actors [25] or seek to change the way policy actors conceptualise an issue [26].

This study lends further weight to a review by Hanney et al. [7], which identified that two of the most important areas influencing research uptake are the interfaces between health research and policy, and the characteristics of policy actors as receptors of research. The research actors we interviewed were actively seeking to build relationships with policy actors in order to strengthen the research-policy interface, but that this involved a number of tensions. Firstly, research actors face challenges in carrying out intensive and continuous policy engagement with limited skill sets and resources. Strategic partnerships may help to address this tension by building on the skills and influence of others [27]. The importance of allocating sufficient time, resources and capacity for research communication needs to be acknowledged by funders and institutions alike [12], and there is a need for research institutions to examine their incentive systems to consider whether and how communication activities can be rewarded as well as academic achievements. Research actors who participated in this study pointed to the risks of negative impact, and the need for communications ethics to be considered as part of research ethics, and for the risks involved to be considered when planning communications work. The study also points to the need to avoid contributing to information overload through non-strategic or inappropriate communications, supporting Walter et al.’s [28] observation that effective communications should ‘analyse the research impact context and target specific barriers to and enablers of change’.

Our findings reveal the variety of views about the role of researchers in policy processes, and the need for this issue to be addressed explicitly in research communication strategies. There are researchers who question the appropriateness of increasing the intensity and variation in research communications activities, although the majority of those we interviewed regarded effective communication as essential and desirable for ensuring that research has an impact. The diverse research approaches and influencing goals of the research actors we interviewed suggest that rather than having a blueprint approach to intensification and diversification of policy engagement for all research actors and institutions, a variety of kinds of engagement and research influencing goals are desirable. Our study suggests that decisions about the appropriate communications strategy should be based in part on the researchers’ discipline and the characteristics of the evidence, as well as analysis of the policy environment and the different links and partnerships available to the research actors. We argue that research actors can play a variety of roles in a continuum from research for knowledge and research for advocacy, and all these roles can be valuable as long as research actors base their communications strategies explicitly on analysis of the uses and limitations of their research evidence, the context they are in, their linkages with those they wish to influence and the skill sets of themselves and their partners.

## Conclusions

In this paper, we carried out a qualitative analysis of experiences of research actors in communicating SRH, HIV and AIDS research evidence. Our findings show that the characteristics of researchers and their institutions, policy context, the multiplicity of actors, and the nature of the research evidence all play a role in policy influencing processes. Research actors perceived a trend towards increasingly intensive and varied communication approaches, yet they face challenges in carrying out intensive and continuous policy engagement with limited skill sets and resources. Even if initiatives to protect university spending on communications, such as the ten percent guideline recommended by DFID and the Economic and Social Research Council (ESRC) of the UK [29], current trends restricting research budgets may indirectly restrict communications resources. Effective influencing strategies include making strategic alliances and coalitions and framing research evidence in ways that are most attractive to particular policy audiences. Research actors experienced tensions including the need to identify and avoid unnecessary communication or unintended impacts, challenges in assessing and attributing impact and the need for adequate resources and skills for communications work.

Our findings highlight the need for researchers and research communicators to use self-reflection when strategising about how to ensure their research is used by their target audiences. We adapted the RAPID framework [11] to include a central sphere, which explicitly focuses on research actors’ positionality in policy processes. We contend that this adapted framework can help researchers and communications specialists to consider their unique combination of attributions and skills, make explicit their influencing goals, and to use this reflection in developing their influencing strategy.

## Competing interests

The authors declare that they have no competing interests.

## Authors' contributions

JC and ST conceptualised and designed the study and carried out interviews and data analysis. JC drafted the paper and ST contributed to drafting and revising the manuscript. Both authors read and approved the final manuscript.
